# The Future of Collateral Artery Research

**DOI:** 10.2174/1573403X113099990001

**Published:** 2014-02

**Authors:** Nazanin Hakimzadeh, Hein J. Verberne, Maria Siebes, Jan J. Piek

**Affiliations:** 1 Department of Biomedical Engineering & Physics,; 2 Department of Nuclear Medicine,; 3 Deptartment of Cardiology, Academic Medical Center, University of Amsterdam, The Netherlands

**Keywords:** Arteriogenesis, angiogenesis, collateral artery growth, coronary arteries, monocytes, non-invasive imaging.

## Abstract

In the event of obstructive coronary artery disease, collateral arteries have been deemed an alternative blood source to preserve myocardial tissue perfusion and function. Monocytes play an important role in modulating this process, by local secretion of growth factors and extracellular matrix degrading enzymes. Extensive efforts have focused on developing compounds for augmenting the growth of collateral vessels (arteriogenesis). Nonetheless, clinical trials investigating the therapeutic potential of these compounds resulted in disappointing outcomes. Previous studies focused on developing compounds that stimulated collateral vessel growth by enhancing monocyte survival and activity. The limited success of these compounds in clinical studies, led to a paradigm shift in arteriogenesis research. Recent studies have shown genetic heterogeneity between CAD patients with sufficient and insufficient collateral vessels. The genetic predispositions in patients with poorly developed collateral vessels include overexpression of arteriogenesis inhibiting signaling pathways. New directions of arteriogenesis research focus on attempting to block such inhibitory pathways to ultimately promote arteriogenesis. Methods to detect collateral vessel growth are also critical in realizing the therapeutic potential of newly developed compounds. Traditional invasive measurements of intracoronary derived collateral flow index remain the gold standard in quantifying functional capacity of collateral vessels. However, advancements made in hybrid diagnostic imaging modalities will also prove to be advantageous in detecting the effects of pro-arteriogenic compounds.

## INTRODUCTION

Chronic coronary artery occlusion due to atherosclerotic plaque progression leads to ischemia distal to the site of obstruction. Although numerous advancements have been made in the treatment of patients with coronary artery disease (CAD), one in five patients is not suitable for revascularization interventions [1]. Nonetheless, chronic ischemia is innately challenged by remodeling of small pre-existing collateral arteries into larger caliber vessels, a neovascularization process known as *arteriogenesis*. Unlike other neovascularization processes such as *angiogenesis*, collateral vessels have the capacity to carry a larger volume of blood than sprouting capillary networks. 

One third of patients with CAD, exhibit a well-developed collateral network. These patients demonstrate better preservation of myocardial function and are less vulnerable to adverse cardiac events with decreased mortality [2-5]. Patients with slowly progressing lesions display better collateral networks, as these vessels have sufficient time for growth and maturation [2]. However, in many cases of rapid plaque progression there is insufficient time for collateral vessel growth, resulting in severe ischemia, hypoxia, necrosis and subsequent scar tissue formation. 

Extensive efforts have focused on understanding the fundamental processes driving collateral artery growth in order to develop pro-arteriogenic compounds. Identification of important inflammatory agents that play a pivotal role in driving collateral vessel formation, with promising preclinical studies paved the path towards initial clinical testing. Unfortunately, these initial trials were met with disappointing conclusions, as alarming side effects of atherosclerotic plaque progression seemed to outweigh the minimal and even negligible therapeutic outcomes [1, 6].

Re-evaluation of molecular and cellular targets with effective clinical endpoints to assess therapeutic outcome is now underway. Conventional thinking surrounding collateral artery research has now been modified to consider the genetic heterogeneity between good arteriogenic responding patients and bad arteriogenic responders [7]. In addition, new methods of non-invasive diagnostic imaging techniques are now in development for improved detection of collateral vessels. Employing multimodal imaging modalities to effectively assess collateral vessel growth is now regarded as a critical means to evaluate the true therapeutic potential of pro-arteriogenic compounds. Although many challenges still remain in translating experimental studies of collateral vessel research towards clinical application, many pivotal advancements have been made in recent years.

## MODULATORS OF ARTERIOGENESIS

The sequence of collateral artery development can be divided into three phases: initiation, growth and maturation. With progressive stenotic expansion, an interplay of physical forces acting as the intial trigger is followed by leukocyte recruitment that modulates the process of arteriogenesis. These triggers are the foundation from which pro-arteriogenic compounds have been derived. An overview of these mechanisms is shown in Fig. (**1**).

### Stimulation by Wall Shear Stress

Chronic stenotic expansion in coronary arteries leads to a sharp pressure drop in the downstream arterial anastomoses. This steep pressure gradient drives increased blood flow through pre-existing bridging collateral vessels, and thereby allowing blood flow to circumvent the site of obstruction. Increased blood flow through collateral arteries leads to a subsequent elevation in local wall shear stress that is detected by endothelial mechanosensors, including caveolae, ion channels, apical glyocalyx, receptor Tyr kinases, primary cilia, heterotrimeric G proteins, intercellular junctions and integrins [8, 9]. In recent years, Tzima *et al*. have shown that endothelial cells (ECs) are also equipped with a mechanosensory complex consisting of platelet EC adhesion molecule (PECAM-1), vascular endothelial (VE)-cadherin, and vascular EC (VEC) growth factor receptor (VEGFR)2 [10]. Endothelial cells exposed to prolonged laminar shear stress, experience configurational changes of cell surface adhesion receptors (including selectins and integrins). These changes result in cytoskeletal remodeling and subsequent activation of signal transduction pathways ultimately leading to an anti-apoptotic state, and increased release of the vasodilator nitric oxide (NO)[11]. Jalali *et al*. have shown that shear stress also increases integrin avidity in ECs [12]. Changes in integrin avidity would likely facilitate leukocyte-EC interaction, a process important in collateral growth propagation. 

It is well recognized that changes in fluid shear stress directly affect leukocyte rolling and tethering [13]. Yago *et al*. demonstrated that at a certain shear threshold, the rolling velocity of leukocytes is reduced while the rate at which they tether to selectins increases [14, 15]. In parallel, slow rolling leukocytes encounter integrins with increased avidity, causing them to come to arrest [13]. This is a critical step in subsequent transmigration to the perivascular space [16]. 

In the absence of shear stress, collateral vessels regress by a process called ‘pruning’, while larger caliber vessels continue to remodel outward even after shear stress has ceased [17, 18]. It is likely that bridging vessels that do not carry a bulk flow degenerate as the endothelium returns to a state of homeostasis due to an inadequately long shear stress exposure. Mathematical simulations of two vessels in parallel, predict that shear stress distribution at the endothelial level does not depict stable collateral vessel growth, as instability promotes the growth of only a few large vessels [19]. These theoretical postulations were later confirmed in experimental studies by Hoefer *et al*. [17]. The authors showed in the ischemic rabbit hind-limb, an initial phase whereby numerous pre-existent arterioles increase conductance within 7 days, followed by a sub-acute phase with a more drastic up-rise in conductance over a period of 3 weeks, driven by the growth of a few large caliber vessels, and a paralleling regression of smaller vessels [17]. Van den Wijngaard *et al*. have also shown that a sub-group of collateral vessels develops in the absence of shear stress, suggesting that areas with localized changes in fluid shear stress result in a global response perhaps by means of subsequently activated circulating molecular and cellular players [20]. 

### Propagation of Growth by Circulating Leukocytes – Role of Monocytes

Stimulation of collateral vessel endothelium by elevations in shear stress, leads to a cascading inflammatory response with infiltrating leukocyte populations. Within the lumen of recruited collateral vessels, shear stress mediates the activation of the transcription factor nuclear factor of kappa light chain gene enhancer in B cells (NF-κB) [21]. Subsequent stimulation of NF-κB-dependent genes follows. This activation involves elevated endothelial expression of adhesion molecules including endothelial selectin (E-selectin), intercellular adhesion molecule (ICAM)-1 and vascular cell adhesion molecule (VCAM)-1 [22, 23]. Woolf *et al*. have shown that leukocytes can only adhere to ICAM1 once increased flow is applied [24]. Upregulation of these adhesion molecules combined with increased avidity (in response to shear stress) facilitates endothelial-leukocyte interaction. Abrogation or blocking of such adhesion molecules leads to impediment of arteriogenesis [25]. 

The specific cocktail of adhesion and chemokine receptors expressed governs which particular leukocyte populations extravasate. Transmigration of monocytes through the endothelium into the perivascular space of activated collateral vessels was initially described in 1976 by Schaper *et al*. [26]. The recruitment of monocytes to these regions occurs in response to the upregulation of the monocyte chemoattractant protein-1 (MCP1) in activated collateral vessels. Demicheva *et al*. recently described that MCP1 expression is initially upregulated in smooth muscle cells (SMCs) in response to changes in circumferential stretching and not wall shear stress [28]. The authors further demonstrated both in a classical hind limb ischemia mouse model and in an ear artery ligation model that MCP1 expression is governed by the mechanosensitive transcription factor activator protein-1. Nonetheless, in response to circumferential changes of the vessel wall, upregulation of the ligand MCP1 results in monocyte infiltration via its respective receptor C-C chemokine receptor 2 (CCR2). Monocytes then adhere to the vessel wall via ICAM1/Mac-1 binding [25], followed by transmigration between endothelial junctions (paracellular migration) or directly through pores in individual endothelial cells (transcellular diapedesis) [27]. 

Following extravasation, monocytes modulate collateral growth by paracrine activity. Monocytes differentiate into macrophages and orchestrate extracellular matrix degradation by secretion of matrix metalloproteinases (MMP), including MMP1-3,7-10, 12 and 14 [29]. In parallel, monocytes recruit other leukocytes as well as modulate SMC and EC proliferation and migration by secretion of stromal derived factor 1 (SDF1), platelet derived growth factor subunit B (PDGFB), tumor necrosis factor α (TNFα ), vascular endothelial growth factor (VEGF) and fibroblast growth factors (FGF1, basic FGF) [30, 31]. Collectively, these result in thickening of the tunica media by SMC proliferation and vessel diameter expansion [32, 33]. 

Nonetheless, monocytes/macrophages represent a heterogeneous population with distinct functional and phenotypical properties. Based on expression levels of cell surface markers, human monocytes can be divided into classical (CD14++CD16-), intermediate (CD14++CD16+) and non-classical (CD14+CD16++) subtypes [34]. Classical monocytes are known to secrete pro-inflammatory cytokines, while non-classical monocytes support an anti-inflammatory environment [35]. The role of intermediate monocytes is still largely debated. In recent studies, Kocaman *et al*. showed that elevated levels of circulating monocytes (without distinction of specific sub-populations) were correlated with good collateral development in CAD patients [36]. Meanwhile, Arslan *et al*. demonstrated that elevated levels of classical monocytes were significantly associated with good collateral development in patients with <95% stenosis in at least one major coronary artery [37].

Once monocytes enter the perivascular space of recruited collateral vessels they differentiate into macrophages. Depending on the environment, macrophages also polarize towards a distinct phenotype (pro-inflammatory M1 or pro-angiogenic M2). M2 macrophages were deemed pro-angiogenic in a tumor angiogenesis study [38]. In relation to arteriogenesis, Takeda *et al*. recently showed that skewed polarization of macrophages towards an M2 phenotype supports collateral artery growth [39]. This particular phenotype of macrophages was driven by deletion of one allele in the oxygen sensor prolyl hydroxylase-2 (PHD2). Haploinsufficiency of PHD2 resulted in an increased level of tissue macrophages at baseline conditions, resulting in a larger pre-existing collateral vessel network. The underlying mechanisms for collateral vessel preconditioning at baseline conditions were attributed to NF-κB activation and M2 secretion of SDF-1 and PDGF-B. Release of these cytokines supported SMC proliferation and migration [39].

The role of other leukocyte populations in arteriogenesis is still relatively unknown. It has been suggested that many leukocytes infiltrate to sites of collateral artery growth in the initial phases and help to recruit monocytes [40, 41]. In numerous inflammatory responses, neutrophils are among the first leukocytes to be recruited to stimulated vessels from the circulation [42]. Infiltration of neutrophils has been noted in the perivascular region of recruited collateral vessels during the initial phases of growth, followed by rapid clearance [42]. Although Hoefer *et al*. suggest that enhanced neutrophil infiltration does not promote arteriogenesis [43], Okhi *et al*. showed that elevated neovascularization by granulocyte colony stimulating factor (G-CSF) administration was attributed to neutrophil secretion of VEGF, leading to progenitor cell mobilization [44]. Similarly, Soehnlein *et al*. demonstrated that secretion products of activated neutrophils stimulate mobilization of classical monocytes, but do not affect extravasation of non-classical monocytes [45]. 

Similar to neutrophils, lymphocyte subsets (CD4+ and CD8+ T cells) have been implicated in aiding monocyte recruitment to activated collateral vessels. This role initially gained attention when impaired arteriogenesis was noted in athymic nude mice, which lack T cells but contain sufficient numbers of monocytes [46]. It has been suggested that infiltrating CD4+ T cells promote collateral growth by secretion of VEGF [47], and CD8+ T cells regulate trafficking of CD4+ T cells and monocytes by interleukin-16 secretion (IL-16) [48]. CD4 knockout mice display reduced capacity of collateral vessel development, which was attributed to reduced VEGF expression and impaired monocyte recruitment [47]. 

Although there are limited studies examining the role of natural killer cells and mast cells in arteriogenesis, both cells have also been implicated in playing a role in the initial phases of collateral vessel growth by modulating inflammatory cell recruitment. It has been suggested that natural killer cells may also aid in tissue clearance during the preliminary expansion phase [40].

## TRIALS OF ARTERIOGENESIS STIMULATION BY MONOCYTE STIMULATION

Extensive efforts have focused on unraveling the complex cascade of events leading to collateral vessel development, with the ultimate goal of identifying potential therapeutic targets. Although steps towards realizing new therapeutic agents for arteriogenic stimulation have been made, these advancements include many short-comings. Numerous compounds targeting monocyte function or endothelial and smooth muscle cell proliferation have shown promising beneficial effects in experimental settings. Among the many compounds identified, MCP1 and colony stimulating factors (CSFs) have been the most widely tested for their ability to enhance monocyte homing and survival. However, therapeutic potential of these compounds in experimental animal models lead to disappointing results in clinical trials. 

## MCP1

**Fig. (2):**
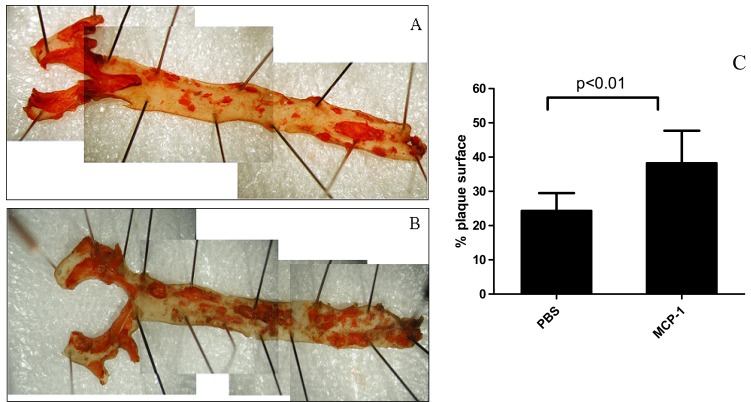
Aortas of ApoE mice with Sudan IV staining (A, PBS; B, high-dose MCP-1). Treatment of mice with MCP1 (10 μg/kg per week) for 2 months lead to an increased percentage of atherosclerotic plaque surface in aortas (C, 24.3 ± 5.2 % for PBS versus 38.2 ± 9.5 % MCP1; p<0.01, n=21). PBS, phosphate buffered saline. Published with permission from Wolters Kluwer Health. Reference [51].

In response to laminar shear stress, collateral arteries dilate. Circumferential stretching detected by SMCs, leads to an upregulation of MCP1 expression [28]. As described, this chemoattractant mediates the recruitment of monocytes to local areas. Numerous groups have shown that systemic infusion of MCP1 enhanced collateral growth in hind-limb ischemia models [17, 49]. Nonetheless, compounds targeting monocyte chemoattraction also pose risks of atherogenesis. Thus, questions arose regarding the effects of local intra-arterial administration of low doses of MCP1 on plaque burden and collateral development. In hyperlipidemic rabbits, intra-arterial infusion of MCP1 did not increase serum lipid levels [50]. However, in other hyperlipidemic animals (Apoe-/- mice) local MCP1 administrations lead to neointima development and increase in plaque surface area relative to controls (Fig. **2**). Changes in pre-existing plaque composition were noted; these changes included decreasing percentage of SMCs and increasing monocyte adhesion in the aortic endothelium [51]. This led to the conclusion that MCP1 while enhancing collateral circulation, also drives atherosclerotic lesions towards a vulnerable plaque phenotype. 

### Colony-stimulating Factors (CSF) 

Granulocyte-macrophage colony-stimulating factor (GM-CSF) and granulocyte colony-stimulating factor (G-CSF) are cytokines released by numerous cells, including endothelial cells in response to laminar flow [52]. Their lucrative function in pro-arteriogenesis applications is their ability to mobilize progenitor cells from the bone marrow, while also promoting survival, proliferation, and differentiation of multiple hematopoietic cell populations, including monocytes [53-55]. Both compounds have shown therapeutic potential in stimulating arteriogenesis in experimental studies.

Intravascular and subcutaneous infusions of GM-CSF have been shown to stimulate collateral vessel growth in the ischemic rabbit hind-limb and in rats with hemodynamic stroke [56, 57]. Contrary to MCP1, GM-CSF demonstrates anti-atherogenic properties by influencing lipid metabolism. Systemic infusion accelerates clearance of LDL in plasma via a LDL-receptor dependent and independent manner [58-60]. Atherosclerotic plaques in hyperlipidemic Watanabe rabbits treated with GM-CSF displayed significant reduction in size as compared to controls [61]. GM-CSF has already been implemented into clinical practice in the treatment of patients suffering from hematologic and oncologic disorders [62, 63]. The anti-atherogenic properties and clinical approval allowed for the accelerated testing of GM-CSF to promote collateral vessel growth in CAD patients. In 2001 Seiler *et al*. initiated the first randomized placebo-controlled trial investigating collateral vessel growth in CAD patients with GM-CSF treatment. Twenty-one coronary artery disease patients received either placebo treatment or intracoronary bolus infusion of 40 μg/kg of GM-CSF, followed by a two week period of subcutaneous injections of 10 μg/kg GM-CSF every other day [64]. Patients treated with GM-CSF demonstrated a significant improvement in collateral artery flow, as determined by pressure-derived collateral flow index measurement. Based on these positive findings, the therapeutic potential of GM-CSF was extended to collateral vessel development in peripheral artery disease. Forty patients with moderate or severe intermittent claudication were treated with subcutaneous injections of GM-CSF (10 μg/kg) or placebo for a two week period, with a total of 7 injections. Although, patients in the treatment group displayed significantly higher walking distance relative to the placebo group at 14 days, the primary end-point of change in walking time showed negligible difference. The lack of therapeutic outcome was attributed to possible differences in the coronary and peripheral circulation, as well as the reduction in dosage administered in this study compared to the study of Seiler *et al*. [64, 65]. Similar to MCP1, safety concerns arose regarding the progression of CAD in response to GM-CSF treatment. Zbinden *et al*. conducted a study with 14 patients investigating the safety and efficacy of subcutaneous GM-CSF infusion in patients with CAD. The study was halted prematurely as two of seven patients in the treatment group suffered an acute coronary syndrome, while none in the placebo group had such an event [1].

Following the disappointing outcomes with GM-CSF trials, attention was focused on G-CSF. In experimental studies with swine, G-CSF has been shown to improve cardiac function and reduce cardiac remodeling following acute myocardial infarction (MI) [66]. Similarly, in a mouse model, subcutaneous administration of 100 μg/kg/day of G-CSF for a period of 5 days after acute myocardial infarction, significantly improved post-MI survival and reduced ischemic cardiomyopathy. The authors also noted G-CSF treatment augmented arteriogenesis, as detected by an increase in ICAM1 expression [67]. Although numerous clinical investigations were initiated to investigate the capacity of G-CSF to prevent adverse left ventricular (LV) remodeling in patients with acute myocardial infarction [68-72], limited studies have tested its application in coronary artery growth. Recently, Meier *et al*. assessed the efficacy of G-CSF treatment for collateral artery growth and its effects on myocardial salvage in 52 chronic stable CAD patients. Patients were administered subcutaneous injections of 10 μg/kg G-CSF or placebo for a period of 2 weeks. In comparison to the control group, the treatment group showed improved signs of myocardial salvage based on the disappearance of ECG ST segment elevation. These improvements were attributed to enhanced collateral vessel function, as measured by pressure-derived collateral flow index [73]. Unfortunately, the use of G-CSF has also raised safety concerns. In a study by Hill *et al*. patients with refractory angina were given subcutaneous G-CSF treatment (5 μg/kg/day) over a 5 day period. Two of 16 patients in the treatment group suffered an acute myocardial infarction, one of which resulted in a fatality [6]. Although, larger clinical studies did not result in increased prevalence of adverse events, future trials were only to commence with greater precautions on safety.

## ARTERIOGENESIS VS. ATHEROGENESIS - THE ‘JANUS PHENOMENON’

**Fig. (3):**
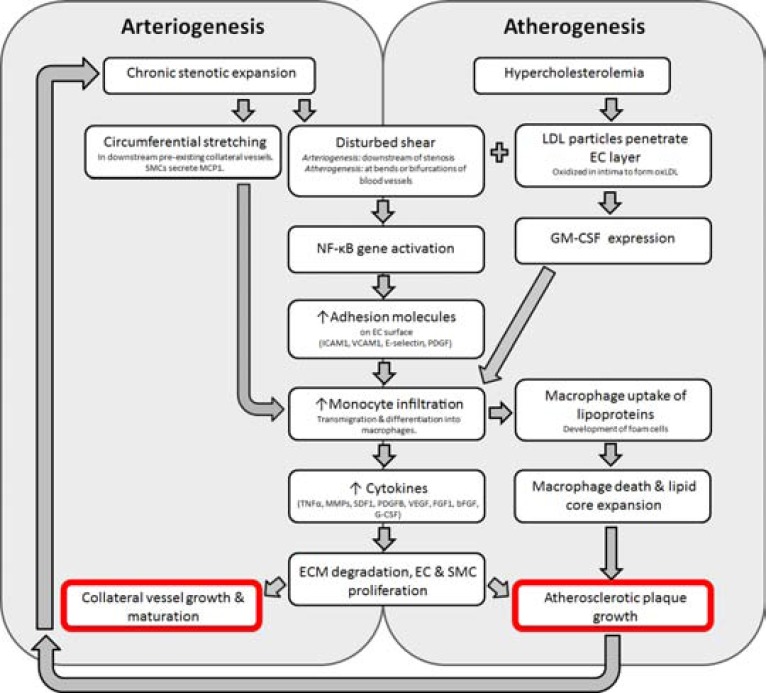
Overlapping pathways common to arteriogenesis and atherogenesis. Collateral vessel formation leads to subsequent circumferential stretching and elevated shear stress in the downstream pre-existing collateral network. This leads to secretion of MCP1 by SMCs, inducing monocyte infiltration. Common to both arteriogenesis and atherogenesis, NF-κB activation in response to disturbed shear leads to increase in adhesion molecule expression on ECs, facilitating EC-leukocyte interaction and monocyte infiltration. Monocytes release pro-inflammatory cytokines influencing ECM degradation, EC and SMC proliferation and thereby facilitating collateral vessel growth and maturation. In the context of hypercholesterolemia, LDL particles accumulate in the intima, leading to the development of oxLDL and thereby stimulating GMCSF secretion. This cytokine facilitates hematopoietic cell mobilization, including monocytes. Transmigration of monocytes to areas rich in lipoproteins, causes them to phagocytose surrounding lipoproteins, leading to the development of foam cells and expansion of the lesion. Growth of atherosclerotic plaques re-trigger the entire process of arteriogenesis. bFGF: basic fibroblast growth factor; EC: endothelial cell; ECM: extracellular matrix; FGF1: fibroblast growth factor 1; G-CSF: granulocyte colony stimulating factor; GM-CSF: granulocyte macrophage colony stimulating factor; ICAM1: intercellular adhesion molecule 1; LDL: low-density lipoprotein; oxLDL: oxidized lowdensity lipoprotein; MCP1: monocyte chemoattractant protein 1; MMP: matrix metalloproteinase; NF-κB: nuclear factor of kappa light chain gene enhancer in B cells; PDGF: platelet derived growth factor subunit B; SDF1: stromal derived factor 1; SMC: smooth muscle cell; TNFα: tumour necrosis factor α; VCAM1: vascular cell adhesion molecule 1; VEGF: vascular endothelial growth factor.

Unwanted side effects existing for any potent therapeutic compound is not uncommon. This benefit vs. risk of arteriogenesis vs. atherogenesis introduces what Epstein *et al*. referred to as the ‘Janus phenomenon’ [74]. Propagation and sustainment of inflammatory cytokines, chemokines, monocyte infiltration and adhesion molecules allowing enhanced endothelial-leukocyte interaction are critical in both arteriogenesis and atherogenesis. The overlapping inflammatory pathways, deems the implementation of any growth factor for collateral vessel growth potentially dangerous for plaque progression (Fig. **3**).

Similar to arteriogenesis, atherogenesis is a flow and shear mediated phenomenon. Atherosclerotic lesions often develop in areas with disturbed flow and shear patterns, which leads to sustained activation of NF-κB, and subsequent stimulation of NF-κB-dependent genes [75]. As described, these genes encode proteins such as ICAM1, VCAM1, E-selectin and PDGF which are also important in arteriogenesis. In parallel, regions susceptible to atherosclerotic plaque development display expression of these molecules in the early stages of lesion growth [23]. 

In animal models, genetic heterogeneity between different strains of mice has shown that animals with good collateral vessel development are also highly susceptible to atherosclerosis. In contrary, mice that are not vulnerable to atherosclerosis, also display poor collateral anastomoses [76, 77]. Genetic heterogeneity leading to such phenotypic differences between robust collateral vessel formers vs. inferior collateral formation, and respective susceptibility to atherosclerosis, suggests possible genetic predispositions [41, 78, 79]. Identification of these genetic predispositions will allow for new mechanistic hypotheses to be explored, such that new pro-arteriogenic targets without possible atherogenic consequences can be developed.

## PARADIGM SHIFT IN ARTERIOGENESIS RESEARCH

Failure of numerous clinical trials made it imperative to change the traditional bench to bedside approach of seeking pro-arteriogenic compounds. The initial clinical trials implemented targets identified in experimental models of collateral artery growth. The subsequent disappointing outcomes led to the initiation of clinical studies with the goal of identifying appropriate factors in CAD patients. It was hoped that these studies may help identify factors causing some CAD patients to have well-developed collateral networks versus others with poor collateral anastomoses. Findings from such studies were then explored in experimental models. This change from the conventional bench to bedside approach is part of the *paradigm shift* in collateral artery research. Such a reversal from bedside to bench tactic may also prove to be relevant and advantageous in other clinical disorders. 

Due to the inaccessibility of human collateral arteries, much remains to be elucidated in human arteriogenesis research. Investigations of signaling pathways modulating collateral artery growth in humans has been attempted in few studies. However, analysis of systemic cytokine levels in plasma samples of patients with varying degrees of collateralization has resulted in inconsistencies [80, 81]. The divergent findings have been attributed to the fact that systemic levels of growth factors are likely different than local cytokine levels at sites of collateral vessel growth. Schirmer *et al*. demonstrated in patients with immature collateral circulation, a larger oxygen gradient, as well as elevated levels of pro-arteriogenic cytokines (eotaxin, bFGF, MCP1, transforming growth factor β and macrophage migration inflammatory factor) relative to patients with a more developed collateral circulation [82]. These findings confirm the importance of seeking specific targets that play a direct role in the confined regions of actively growing collateral vessels.

Nonetheless, to identify appropriate targets and elucidate genetic heterogeneity between patients with varying degrees of collateralization, local plasma samples are not sufficient and cumbersome to obtain. Transcriptional profiling of circulating monocytes, which play a large role in the local process of collateral growth, was identified as the most easily attainable and logical source. Monocytes can be easily extracted from the peripheral blood, and are a reflection of the local processes of collateral artery growth. Numerous studies have confirmed that the response of monocytes in the systemic circulation is a good reflection of the local processes of arteriogenesis [36, 37, 83]. 

Chittenden *et al*. initially sought to identify molecular markers characteristic of a “noncollateralgenic” phenotype in CAD patients [84]. Sixteen patients were divided into two groups of 8 based on angiographic assessment of collateral circulation. Peripheral blood monocytes were obtained and underwent transcriptome analysis. The authors stated that circulating monocytes of patients with poorly developed collateral arteries, had increased expression of apoptotic genes, and decreased expression of cell proliferation genes. Chittenden *et al*. also concluded that these distinct transcriptional profiles between good and bad collateral circulation patients was independent of CAD severity or other known clinical parameters that may affect collateral vessel development [84]. Similarly in another study by Meier *et al*., consisting of a larger cohort of patients (110 CAD patients) and also 50 individuals without CAD, attempts were made to identify genetic markers that are characteristic of a well-developed collateral network [85]. Patients were deemed as having well-developed or insufficient collateral network based on pressure-derived collateral flow index (CFI_p_) measurements. The authors conducted transcriptional profiling of un-stimulated peripheral blood monocytes, and monocytes stimulated with MCP1 from the respective groups. The authors showed that monocytes from patients (with or without CAD) with poor collateral network have different gene expression patterns and also display a weaker response to MCP1 [85].

**Fig. (4):**
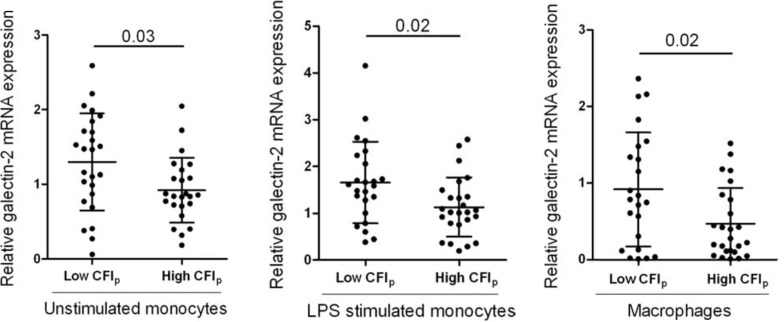
Patients with insufficient collateral network (ie. low CFIp) show elevated levels of galectin-2 mRNA expression in different monocyte phenotypes (n= 25 vs. 25, data shown as mean ± SD). CFIp: pressure-derived collateral flow index; LPS: lipopolysaccharide. Published with permission from Oxford University Press. Reference [7].

In a larger clinical study by Schirmer *et al*., transcriptional profiling of circulating monocytes from patients with either poor or well developed collateral circulation revealed 244 differentially expressed genes [86]. Taking a closer look at the specific pathways showing varying activation levels, Schirmer *et al*. revealed that genes related to type I interferon, primarily interferon-β were overexpressed in patients with poorly developed collateral circulation [86]. Interferon-β mRNA expression levels were elevated in 3 of 4 cellular phenotypes of non-responders, including lipopolysaccharide (LPS) stimulated monocytes. The authors further confirmed the inhibitory effects of interferon-β on collateral formation in a hind-limb ischemia mouse model with systemic administration of interferon-β. Enhanced interferon-β expression was deemed to prevent maturation of collateral vessels by attenuating smooth muscle cell proliferation [87]. Similarly, in a subsequent clinical study RNA extraction from peripheral blood monocytes in 50 patients with obstructive coronary artery disease identified galectin-2 as a novel target in arteriogenesis modulation [7]. Patients that displayed low capacity of collateral circulation showed greater galectin-2 mRNA expression in peripheral blood monocytes (Fig. **4**). In addition, these ‘non-responding’ patients displayed the presence of rs7291467 polymorphism which was associated with elevated galectin-2 mRNA expression and poor arteriogenic response. Systemic infusion of galectin-2 resulted in inhibition of arteriogenesis in a hind-limb ischemia mouse model by modulation of monocyte/macrophage responses.

Collectively, these studies highlight more specific inhibitory pathways that can be targeted rather than stimulatory pathways targeting monocyte/macrophage function. This is the essence of the paradigm shift in arteriogenesis research. Previous pro-arteriogenic attempts focused on augmenting stimulatory pathways. However, in patients that display poor pre-existing collateral networks, it may be more beneficial to block the inhibitory pathways that are likely impeding innate collateral vessel growth. By blocking inhibitory pathways in CAD patients, it is hoped that this will directly result in arteriogenesis stimulation. Recent identification of microRNA (miRNA) has opened a potentially new direction in pharmaceutical development. MiRNA are small non-coding RNA (~22 nucleotides in length) that regulate gene expression at a post-transcriptional level through translational suppression or degradation of downstream mRNA targets [88]. Although *in vivo* data on the role of miRNA in vascular remodelling are still limited and yet to emerge [89], this may be a new direction to pursue in blocking arteriogenesis inhibiting pathways.

These clinical studies also demonstrate the relevance of translating clinical findings to experimental application. In addition, these investigations have shown that factors limiting coronary collateral growth in patients also inhibit collateral vessel growth in the hind limb of rodents, thereby emphasizing the overlap of arteriogenesis progression in the coronary and peripheral circulation. By means of targeting very specific signaling pathways in patients that display poor arteriogenic capacity, rather than enhancing general monocyte/macrophage growth capacity, it may also be possible to avoid the atherogenic properties of pro-arteriogenic compounds. 

## MODE OF ADMINISTRATION OF PRO-ARTERIOGENIC COMPOUNDS

Due to the potential adverse systemic effects of some pro-arteriogenic compounds and in order to maximize therapeutic potential, the mode of administration and dosage of arteriogenic compounds is of critical importance. Local intra-arterial delivery of pro-arteriogenic compounds over a prolonged period of time displays greater efficacy than other modes of administration, including intravenous, intramuscular, subcutaneous or intrapericardial infusion [90-92]. 

In a study by Grundmann *et al*. [92], a direct comparison was made between slow intra-arterial elution of transforming growth factor (TGF)-β1 by stent elution with a single intra-arterial bolus injection of the same dosage of TGF-β1 in a rabbit hind limb ischemia model. Implantation of TGF-β1-eluting stent almost doubled collateral conductance relative to a single bolus infusion of the same dose of TGF-β1, which displayed negligible effects on collateral artery growth. In addition, TGF-β1-eluting stents induced only localized effects as opposed to systemic increases in TGF-β1 plasma levels [92]. This study highlights that the exposure time for some pro-arteriogenic compounds can lead to varying therapeutic effects, even with equal doses.

Studies achieving direct infusion of pro-arteriogenic compounds into the donor artery of the developing collateral circulation have demonstrated greatest therapeutic efficacy. Nonetheless, this mode of administration is not easily attainable in cases of obstructive CAD. Many clinical studies testing pro-arteriogenic compounds have employed intra-arterial bolus infusion, subcutaneous administration, intra-venous injection, or combinations of these methods [64, 65, 93]. These inferior methods of delivery may have been critical limiting factors contributing to the poor outcomes in the clinical studies completed to date. 

An alternative means of achieving local delivery of pro-arteriogenic compounds is by ultrasonic destruction of loaded microbubbles [94]. Microbubbles comprised of albumin or lipids are loaded with a compound (genetic constructs, proteins or cells) and administered predominantly by intravenous injection. Circulation of the microbubbles is tracked by ultrasound, and selective ultrasonic pulsation allows for vehicle destruction and subsequent release of compounds in the region of interest [95]. By loading the shell of microbubbles with antibodies or peptides with affinity for specific ligands, they can bind to specific cells or tissues expressing the respective ligand. Leong-Poi *et al*. targeted actively growing collateral vessels in the ischemic hind-limb of rats by incorporating echistatin (with affinity for alpha(v) and alpha5beta1-integrins) into microbubbles [96].

## DETECTION OF COLLATERAL GROWTH

Further challenges in driving experimental use of pro-arteriogenic compounds towards clinical application are due to the lack of sufficient and reliable means of assessing myocardial collateral perfusion. 

### Traditional Invasive Diagnostic Techniques

#### Coronary Angiography

Coronary angiography has been used in many initial clinical studies for detection of spontaneously visible collateral vessels. Important functional significance has been linked to ‘recruitable’ collateral arteries to prevent ischemic damage and left ventricular dysfunction during short-lived coronary artery occlusion [97]. Angiography can be used to determine the functional capacity of the collateral anastomoses in cases of total chronic coronary artery occlusion (CTO; chronic total occlusion). Werner *et al*. confirmed this in a study of 100 patients with CTO of a major coronary artery present for at least 2 weeks [5]. The authors showed that angiographic grading of collateral vessels was sufficient to determine their functional capacity to preserve regional left ventricular function. In addition, such categorization can be linked to invasively determined parameters of collateral hemodynamics [5, 98]. Nonetheless, vessels smaller than 100µm are left undetected when examined with angiography [99]. In a transluminal coronary angioplasty model with 16 patients, Rentrop *et al*. revealed that collateral vessel visualization is dependent on the respective pressure gradient imposed on the collateral circulation [97]. Based on these findings it was concluded that previous classifications of the extent of collateral vessel growth in patient studies were incorrect. Thus, while coronary angiography is readily available, quantitative assessment of the collateral circulation is limited by its resolution and poor accuracy.

#### Coronary Collateral Vessel Pressure and Velocity Measurements

**Fig. (5):**
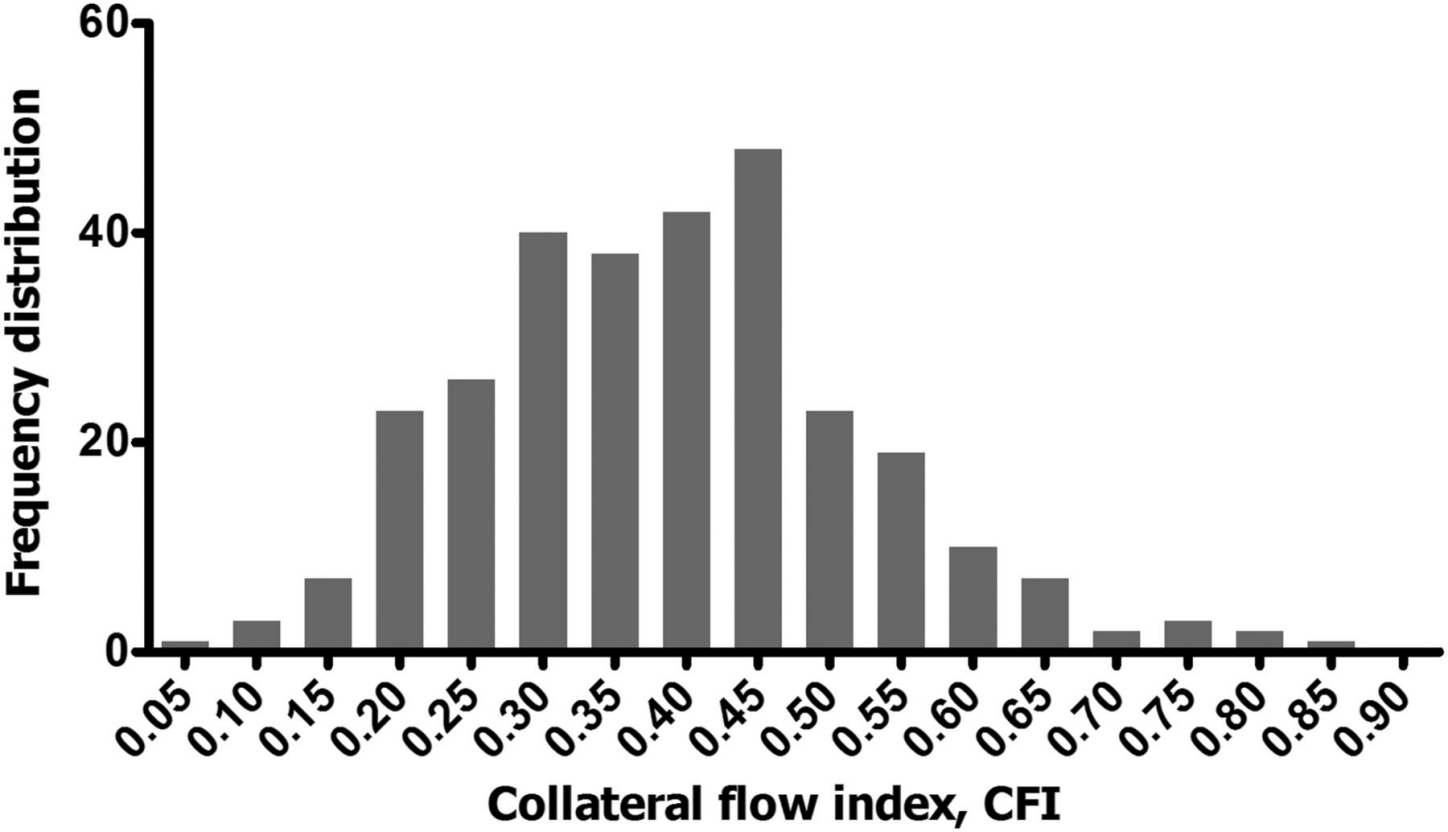
Frequency distribution of pressure-derived collateral flow index (CFI, x-axis) measurements in 295 patients with a chronic total occlusion (CTO), showing Gaussian distribution. In this patient population, the target vessel for percutaneous coronary intervention (PCI) was 34.0% in the left anterior descending (LAD), 46.0% in the right coronary artery (RCA) and 19.0% in the right circumflex (RCX). Frequency distribution shown on Y-axis represents absolute numbers. Published with permission from BMJ Publishing Group Ltd. Reference [102].

The current gold standard for quantitative assessment of the human coronary collateral circulation is by invasive cardiac examination. Flow and pressure measurements obtained by the introduction of ultrathin guidewires equipped with Doppler crystal and pressure sensors allows for quantification of collateral hemodynamics. Simultaneous assessment of aortic pressure, intracoronary velocity and pressure distal to a stenosis during coronary angioplasty in CAD patients, allowed for the derivation of *pressure-derived collateral flow index (CFI_p_)* and *velocity-derived collateral flow index (CFI_v_)* [100]. To obtain such indices, coronary pressure must be initially measured distal to the stenosis during complete balloon occlusion. The more developed the collateral network, the higher the distal pressure during balloon occlusion and the closer the CFI_p_ value approaches 1. CFI measurements in 100 patients without stenotic lesions (or with partial presence of stenotic lesions) revealed a normal distribution of CFI values, with the identification of a group of patients with reference CFI values that represent well-developed collateral vessels [101]. In studies with CTO, whereby the variability of coronary lesion severity is eliminated, a near Gaussian distribution pattern of CFI_p_ is also seen (Fig. **5**) [102], further supporting the notion that genetic predispositions play a role in collateral vessel development. To distinguish between good and bad arteriogenic responders, CFI_p_ measurements with the definition of myocardial ischemia (ST-segment elevation ≥ 0.1mV) have established a threshold of 0.215 [103]. Based on this criterion recent efforts have focused on identifying the innate factors that impact the development of sufficient and insufficient collateral networks.

#### Novel Non-invasive Diagnostic Imaging

Advancements in hybrid imaging modalities, with improved resolution and sensitivity have introduced new possibilities for non-invasive diagnostic imaging. These modalities include magnetic resonance (MR) imaging, computed tomography (CT), positron emission tomography (PET) and single photon emission computed tomography (SPECT). Quantitative assessment of regional myocardial perfusion of collateral blood flow-dependent myocardium in CTO patients can be assessed with non-invasive diagnostic techniques, such as PET, SPECT and MRI. In the cases of non-CTO patients, traditional invasive measurements are important, since without the presence of a natural or artificial occlusion of the collateral receiving artery, blood flow perfusing the downstream vasculature cannot be distinguished from the native or collateral network [104].

Among these non-invasive diagnostic imaging systems, MRI has been deemed as having the greatest versatility with regards to vascular imaging due to its capacity to obtain morphologic and functional information [105]. MRI has the capacity to visualize vessel growth at varying spatial and temporal scales, with greater sensitivity to small vessel function than other imaging modalities [106]. These capabilities could prove to be advantageous for collateral vessel detection.

Nuclear imaging techniques such as PET and SPECT allow the visualization and quantification of the distribution of exogenously administered radioactive isotopes. ^13^N-ammonia and ^15^O-water are used in conjunction with PET imaging in routine clinical practice for the visualization of myocardial perfusion [107]. Visualization and quantification of changes in myocardial blood flow in CAD patients by means of PET offers superior sensitivity with moderate specificity [108]. 

Nonetheless, while some pro-angiogenic or arteriogenic clinical trials have employed SPECT, PET or MRI for perfusion assessment as a means to quantify the therapeutic outcome of stimulatory compounds [109], a new emerging direction is molecular imaging. The vast insight acquired about the signaling pathways and specific modulators of arteriogenesis can be exploited to image the expression of specific molecules. To achieve this, molecules with specific affinity can either be labeled with radioligands or contrast agents. In the case of MRI studies a larger compound is needed, consisting of a nanoparticle and an antibody fragment or ligand with specific affinity for the target molecule [108]. The subsequent size of the imaging agent is also of relevance as it directly impacts extravasation capacity [110]. 

To date, a number of ligands and respective target molecules have been identified for molecular imaging of angiogenesis, some of which are also relevant for arteriogenesis. Perhaps one of the most widely studied molecular imaging agents is the RGD peptide targeting α_v_β_3_. Expression of this integrin is found in activated endothelium of angiogenic vessels, and is undetected in quiescent vessels [111, 112]. Recently, expression of α_v_β_3 _has also been linked to actively growing collateral vessels. Cai *et al*. showed in a recent study that α_v_β_3_ and α_5_β_1_ expression is upregulated in smooth muscle cells of actively growing collateral vessels [113]. Other compounds targeting solely collateral arteries have also been identified by Mazur *et al*. using single chain antibodies. The authors developed collateral-targeting single-chain antibodies that homed specifically to collateral endothelium and not control vessels or angiogenic (tumor) vessels [113]. 

Ultimately, by combining the noninvasive nuclear imaging modalities described (PET or SPECT) with molecular targets, improvements in spatial resolution may be achieved. In addition, multimodal techniques can be used to obtain highly sensitive detection of tracer distribution by means of PET or SPECT, while MRI will reveal complementing functional and anatomical information [114].

## CONCLUSION

Although the beneficial impact of recruitable collaterals was highly debated at one time, it has been well documented now that a well-functioning coronary collateral circulation is important in preventing mortality in patients with chronic stable CAD [3, 115]. Genetic predispositions leading to heterogeneity in the collateral anastomoses has been noted in CAD patients. Transcriptional profiling of monocytes has revealed distinct inhibitory pathways that are overexpressed in CAD patients with poor collateral networks. New efforts must focus on finding means to block these inhibitory pathways, such that innate collateral vessel development can proceed. Previous attempts focused primarily on developing pro-stimulatory compounds for enhancing arteriogenesis. Nonetheless, the short-comings of such compounds seen in clinical trials also highlighted the danger of atheropotency of pro-stimulatory compounds. Patients genetically predisposed to higher expression of inhibitory pathways will likely not respond to pro-stimulatory compounds, as Meier *et al*. [85] showed that their monocytes are less responsive. 

Realization of the therapeutic potential of new strategies to stimulate arteriogenesis requires effective methods of collateral detection. Although, invasive measurements such as CFI_p_ remain the gold standard in CAD patients, non-invasive diagnostic imaging techniques are applicable in CTO patients. Novel advancements made in diagnostic imaging modalities with improved sensitivity and the development of molecular imaging agents have expanded the techniques available for assessing collateral dependent territories in obstructive coronary syndromes. Integration of these techniques with the use of compounds catered to the genetic heterogeneity of CAD patients, will hopefully lead to the long awaited clinical implementation of therapeutic artergiogenesis.

## Figures and Tables

**Fig. (1):**
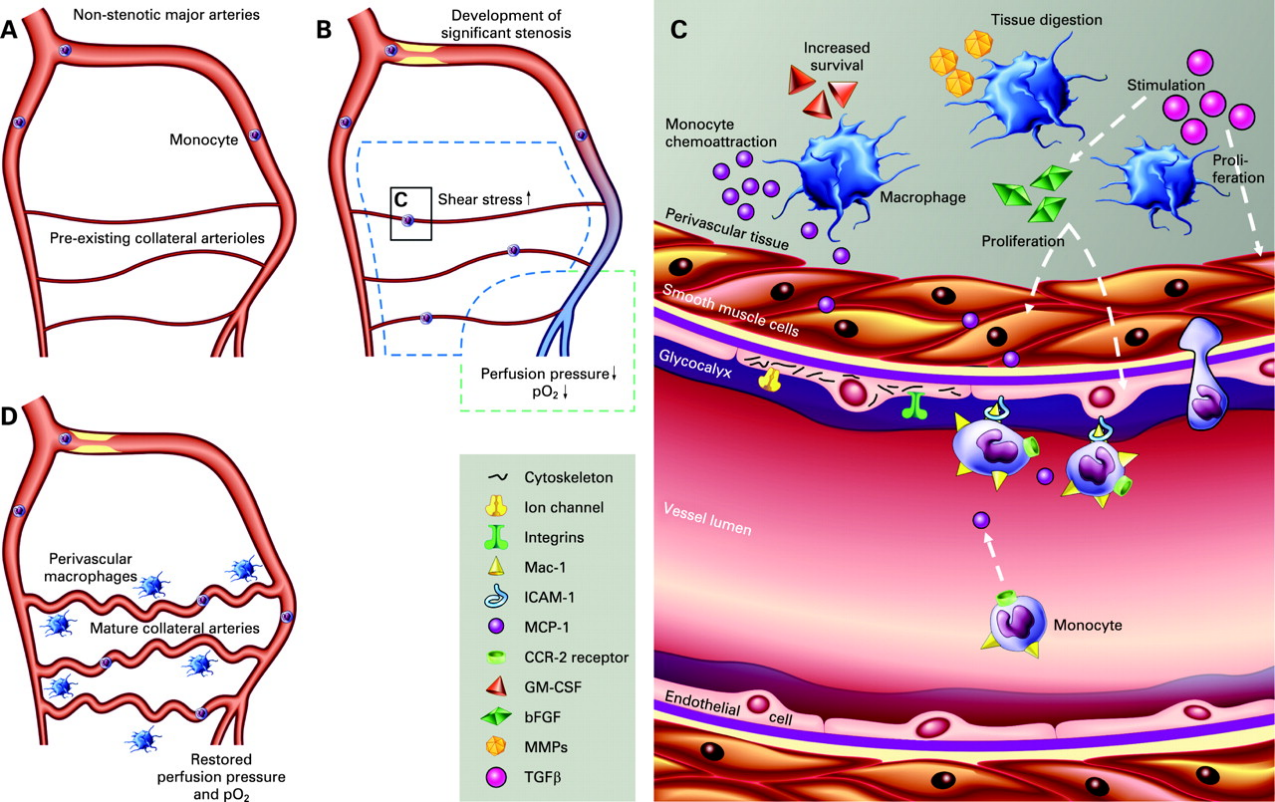
Cellular and molecular mechanisms of arteriogenesis. (A) In the absence of stenosis, pre-existing collateral vessels are small in diameter, carrying minimal blood flow. (B) Chronic stenotic expansion induces a drop in pressure and oxygen saturation in the distal vascular anastomoses (purple colour). Pressure and oxygen saturation in the proximal vascular bed remain unchanged (red colour). This induces a steep pressure gradient over bridging collateral vessels and a subsequent elevation in fluid shear stress. (C) At the cellular and molecular level in activated collateral vessels, endothelial cells respond to changes in shear stress with mechanosensors including transmembrane proteins (integrins, ion channels) and the glycocalyx, resulting in cytoskeletal reorganization and activation of signal transduction pathways. Circumferential stretching and elevated shear stress leads to upregulation of MCP1 in smooth muscle cells and expression of adhesion molecules (including ICAM1) on the surface of endothelial cells. Circulating monocytes expressing CCR2 are recruited to these regions by detection of MCP1 and subsequent binding to the vessel wall by means of ICAM-1/Mac-1 binding. Recruited monocytes transmigrate to the perivascular space where they differentiate into macrophages and modulate smooth muscle cell and endothelial cell proliferation, as well as secreting extracellular matrix degrading enzymes (MMPs). (D) Mature collateral vessels carry a larger blood volume and thereby restore perfusion pressure and oxygen saturation in adjacent vessels distal to the atherosclerotic lesion. bFGF: basic fibroblast growth factor; CCR2: C-C chemokine receptor 2; GM-CSF: granulocyte-macrophage colony-stimulating factor; MCP1: monocyte chemoattractant protein 1; MMP: matrix metalloproteinases; TGFβ: transforming growth factor β. Published with permission from BMJ Publishing Group Ltd. Reference [9].

## ABBREVIATIONS

bFGF: = basic fibroblast growth factor
CAD: = coronary artery disease
CCR2: = C-C chemokine receptor 2
CFI_p_: = pressure-derived collateral flow index
CFI_v_: = velocity-derived collateral flow index
CSF: = colony stimulating factor
CT: = computed tomography
CTO: = chronic total occlusion
EC: = endothelial cell
E-selectin: = endothelial selectin
FGF: = fibroblast growth factor
G-CSF: = granulocyte colony stimulating factor
GM-CSF: = granulocyte macrophage colony stimulating factor
ICAM: = intercellular adhesion molecule
LAD: = left anterior descending
LVEF: = left ventricular ejection fraction
LV: = left ventricular
LPS: = lipopolysaccharide
MCP1: = monocyte chemoattractant protein 1
miRNA: = micro RNA
MMP: = matrix metalloproteinase
MRI: = magnetic resonance imaging
mRNA: = messenger RNA
NF-κB: = nuclear factor of kappa light chain gene enhancer in B cells
NO: = nitric oxide
PDGFB: = platelet derived growth factor subunit B
PECAM1: = platelet endothelial cell adhesion molecule
PET: = positron emission tomography
PHD2: = prolyl hydroxylase-2
SDF1: = stromal derived factor 1
RCA: = right coronary artery
RCX: = right circumflex
SMC: = smooth muscle cell
SPECT: = single photon emission computed tomography
TGF: = transforming growth factor
TNFα: = tumor necrosis factor α
VCAM: = vascular cell adhesion molecule
VE-cadherin: = vascular endothelial cadherin
VEC: = vascular endothelial cell
VEGF: = vascular endothelial growth factor
VEGFR: = vascular endothelial growth factor receptor
